# Comparative metabolomic analysis of polyphenic horn development in the dung beetle *Onthophagus taurus*

**DOI:** 10.1371/journal.pone.0265222

**Published:** 2022-03-17

**Authors:** Naomi G. Williamson, Callee M. Walsh, Teiya Kijimoto

**Affiliations:** 1 Division of Plant and Soil Sciences, West Virginia University, Morgantown, West Virginia, United States of America; 2 Shared Research Facilities, West Virginia University, Morgantown, West Virginia, United States of America; University of Arkansas, UNITED STATES

## Abstract

Organisms alter their phenotypes in response to changing environmental conditions. The developmental basis of this phenomenon, phenotypic plasticity, is a topic of broad interest in many fields of biology. While insects provide a suitable model for studying the genetic basis of phenotypic plasticity, the physiological aspects of plasticity are not fully understood. Here, we report the physiological basis of polyphenism, an extreme form of phenotypic plasticity by utilizing a dung beetle species, *Onthophagus taurus*. We highlighted the metabolome between sexes as well as two distinct male morphs—large and small horns. Unlike results from previous transcriptomic studies, the comparative metabolomic study revealed that differences in metabolite level were more prominent between animals with different body sizes than different sexes. Our results also indicate that specific metabolites and biochemical pathways may be active during horn size determination.

## 1 Introduction

Phenotypic plasticity is a phenomenon in which organisms alter their phenotypes in response to changes in the surrounding environment without altering their genotypes [1–3]. Beetle horns are recognized as a good model for studying the genetic basis of phenotypic plasticity due to their prominent phenotypes and sensitivity to environmental conditions. For instance, multiple reports suggest that the same genetic mechanisms, such as the gene *doublesex* (*dsx*) and the insulin/IGF signaling pathway, are responsible for horn development in different beetle species [4–8].

The dung beetle species *Onthophagus taurus* provides a unique opportunity to study plastic horn development. In this species, horn development is polyphenic, where horn size drastically and almost discretely changes at a certain body size (threshold body size). Specifically, when raised under suboptimal nutritional conditions during the larval stage, animals stay below the threshold body size, and males develop short, almost rudimental horns. Conversely, animals raised under optimal nutritional conditions exceed the threshold body size and develop disproportionately large horns (S1 Fig). Here, the horn size distribution is bimodal, whereas the body size exhibits a normal distribution [9]. Horn development and subsequent horn size determination take place in the larval period, specifically, during 48 hours prior to pupation that is known as the prepupal stage. Cells in the head region proliferate and grow very rapidly to form horn primordia in this stage.

Thus far, two developmental processes and their associated genes have been identified as responsible for both horn development itself as well as polyphenism in *O*. *taurus*: the gene *doublesex* (*dsx*) and genes in the Hedgehog pathway including *hedgehog* (*hh*), *smoothened* (*smo*), and *patched* (*ptc*). In both cases, these genes play critical roles in their original context; sex determination is regulated by *dsx* [10] and axis patterning is regulated by the Hh pathway [11–13]. Additionally, these genes regulate nutrition-dependent horn polyphenism either below (Hh pathway) or above (*dsx*) the threshold body size [4, 14].

Although involvement of the insulin signaling pathway or studies of juvenile hormone titer suggest the presence of certain critical metabolites in horn size determination [15–17], the actual biochemical processes or metabolites that may contribute to the activation and/or suppression of these genetic processes remain relatively unknown. To obtain knowledge regarding the physiological status that may associate with horn polyphenism in *O*. *taurus*, we highlighted the metabolic profiles in different body sizes and sexes, namely large males (LM), small males (SM), large females (LF), and small females (SF). We obtained the first comprehensive list of metabolites that showed significant changes across different body sizes. We also identified candidate metabolites and possible biochemical pathways that may associate with polyphenic horn development. Furthermore, our results suggest that unlike transcriptome studies in the past, body size (determined by nutritional condition) showed greater changes in metabolome pattern than sex.

## 2 Materials and methods

### 2.1 Beetle rearing

Beetles were collected at WVU’s Animal Sciences Farm in Morgantown, WV and used for breeding. Cow dung was collected at the farm and homogenized to ensure beetles were reared under similar nutritional conditions. Larvae were transferred from their brood balls to 12-well plates with similar amounts of homogenized dung and kept at 26 °C in a 16-hour light and 8-hour dark cycle [4].

### 2.2 Sampling

During the prepupal stage, animals stop feeding and purge their gut contents to prepare for pupal development. After they pupate, their external phenotypes, including horn size, are comparable to their adult phenotypes. Thus, to obtain basic knowledge of metabolite composition during horn development, as well as acquire a list of candidate metabolites that may represent the physiological processes during polyphenic horn development, we utilized hemolymph from animals in their late prepupal stage.

Larval development was monitored in 12-well plates, where animals were staged and sexed at appropriate points in their development. Body mass was measured when the larvae reached the prepupal stage. Animals were collected around 48 hours after they entered the prepupal stage (referred as PP2 hereafter). Animals were rinsed briefly in 70% methanol, flash frozen in liquid nitrogen, and stored at -80°C until they were used for hemolymph extraction. Five animals were used in this study for each sample group: Large males (0.1458, 0.1477, 0.1485, 0.1491, 0.1492 g), small males (0.0773, 0.0905, 0.0934, 0.1003, 0.1008 g), large females (0.1374, 0.1430, 0.1435, 0.1469, 0.1568 g), and small females (0.0885, 0.0900, 0.0925, 0.0954, 0.1032 g). The distribution pattern of body mass can be found in S2 Fig.

### 2.3 Methanol extraction of organic compounds from beetle hemolymph

Organic compounds were extracted from hemolymph using a cold methanol extraction method based on Want et al. due to its efficacy and ability to extract a broad spectrum of metabolites [18, 19]. Beetles at PP2 were kept on ice during the extraction process. To extract hemolymph, a small hole was made at the bottom of a 1.7ml microcentrifugation tube (referred to as the sample tube hereafter), containing the stored beetle samples. The sample tube was then placed on top of another 1.7ml tube for collection (referred to as the collection tube hereafter). Hemolymph was obtained by centrifugation at 6,200 x g for 15 minutes at 4°C. Sample tubes that contained large pieces of beetle cuticles were removed from the collection tubes, and the remaining hemolymph in the collection tube was processed further. The soft pellets in the collection tubes were resuspended and centrifuged again for 15 minutes at 17,000 x g to fully pellet insoluble debris and ensure the extraction of compounds. From each sample, equal volumes (30 μl) of supernatant were transferred to the extraction tubes. Ice cold methanol containing 50 μM caffeine and 50 μM each of deuterated amino acids, serine D3 and lysine D9, as internal standards was added in a 3:1 volume ratio to each hemolymph sample. Five quality control (QC) samples were prepared that contained equal amounts of pooled hemolymph from each beetle and were extracted in methanol containing internal standards at the same ratio described above. Samples were then sonicated for five minutes on ice in a water bath sonicator and transferred to a -20°C freezer for one hour. Samples were then centrifuged for 15 minutes at 17,000 x g at 4°C, and supernatants were retained for LC-MS analysis.

### 2.4 Liquid chromatography-mass spectrometry

Liquid chromatography mass spectrometry (LC-MS) was performed using HILIC chromatography (Phenomenex Luna column, 100 mm x 2 mm in dimension, 3 μm particle diameter, 200 Å pore size) on each beetle and QC sample. Additionally, data from blank samples were collected by injection of blank methanol. For all samples, 10 μL were injected into the LC-MS system. Gradient elution was performed using an Accela UHPLC (Thermo Fisher Scientific, Waltham, MA). The aqueous mobile phase consisted of 10mM ammonium acetate in water (mobile phase A), and 100% acetonitrile as the organic mobile phase (mobile phase B). The 35-minute gradient pattern consisted of the following: 5% A held for 1.5 minutes, 5% to 80% A increased over 18.5 minutes, a further increase from 80% A to 90% A over 3 minutes, a decrease from 90% A to 5% A over 2 minutes, and then held constant at initial conditions for 10 minutes. The column temperature was held constant at 35°C. A Q Exactive Orbitrap mass spectrometer (Thermo Fisher Scientific, Waltham, MA) was used to collect full scan MS data for all individual sample, blanks, and QC samples in negative ion mode from m/z 100 to 1000 using 70,000 resolution. Separate, data-dependent MS/MS data files were collected from the QC samples using the same liquid chromatography conditions except with collection of mass spectrometry data for the top 5 most intense peaks over a m/z 100 to 1000 scan range using 17,500 resolution, a 4 m/z isolation window, and 30 normalized collision energy. Dynamic exclusion was set at 10 seconds. Electrospray ionization source conditions consisted of spray voltage of 3.5 kV, sheath gas flow rate of 20 arbitrary units, auxiliary gas flow rate of 8 arbitrary units, and sweep gas flow rate of 1 arbitrary unit. The capillary temperature was set at 320°C, and the S-lens RF level was set at 30.

### 2.5 Untargeted metabolomics data processing and metabolite annotation

Initial review of the raw data files indicated that LC-MS data collection was of acceptable reproducibility. The relative standard deviation of the peak areas of spiked internal standards within the QC samples was used to determine initial data quality and reproducibility, and was found to be less than 15%, which exceeds minimum requirements for untargeted metabolomics studies [20]. Raw data files were further processed by Compound Discoverer (version 3.10.305, Thermo Scientific) using the untargeted metabolomics workflow with statistics to align peaks across data files and pick significant peaks. All raw files from individual insects from the four groups (large male, small male, large female, and small female), five QC samples, and blank injection samples were included in the initial analysis except for one large male sample. During the sample preparation process (above), one of the large male samples was misplaced and was excluded from the analysis.

In subsequent analyses, statistical comparisons were also made between large and small beetles, excluding factor of sex, as well as between male and female beetles, excluding the size factor. Compound Discoverer settings, in all cases, consisted of 10 ppm mass tolerance peak picking of parent m/z values, a minimum S/N threshold of 3, minimum peak intensity of 1 x 10^6^, default setting of 2-minute RT shift for the compound detection parameter, grouping compounds within 0.2-minute RT window, and normalization of peak areas across samples by the constant mean algorithm. Peaks were retained in the data set that exhibited at most a peak area relative standard deviation of 30% within the QC samples. Database searching of ChemSpider (chemspider.com) and mzCloud (mzCloud.org) was performed through Compound Discoverer for compound annotation.

Metabolite features that showed statistically significant differences (p-value < 0.05) between all pairwise comparisons of four sample sets (large male, small male, large female, and small female) at any fold change are enumerated in Table 1. All compound formulas obtained from these analyses are listed in S1 Table, and their annotation is classified as level 3, tentative structures [19].

**Table 1:** Number of metabolites that showed significant differences through pairwise comparisons.

Comparison	Total metabolite number	Higher in first group	Higher in second group
LM/SM	140	105	35
LM/LF	30	28	2
LM/SF	84	67	17
LF/SF	40	20	20
LF/SM	44	22	22
SF/SM	21	14	7

LM: large male, SM: small male, LF: large female, SF: small female.

We considered compounds that had parent m/z values matching compounds in ChemSpider and those with MS/MS spectral matches to compounds within the mzCloud database for further discussion in Table 2. Annotation using both parent m/z values and MS/MS database matching are designated as level two, putative identifications [19].

**Table 2:** Six metabolites were discussed in detail in this research. Highlighted metabolites were only different between the large and small male comparison.

Name of metabolite	Confirmation method/database *	Comparison	Respective relative fold differences	Respective relative p values
D-Xylonic acid	1	SM>LM, SF>LF	5.78, 3.73	0.0190, 0.0379
N-acetyl-DL-glutamic acid	1, 2	SM>LM	1.82	0.0215
Citric acid	1, 2	LM>SM	1.53	0.0345
Glycerol 3-phosphate	1, 2	LM>SM	2.11	0.0356
N-Acetyl-L-glutamine	1, 2	LM>SM, LM>LF, LM>SF	2.36, 2.04, 2.87	0.0011, 0.0227, 0.0011
D-Glutamine	1, 2	LM>SM, LM>LF, LM>SF	1.57, 1.41, 1.48	0.0052, 0.0418, 0.0347

* 1: ChemSpider, 2: mzCloud (ms/ms).

### 2.6 Hierarchical clustering and principal component analysis

A hierarchical model with Pearson distance function and complete linkage method was chosen to compare the relative metabolite enrichment across samples. We utilized the Compound Discoverer suite with the setting “scale before clustering” for the analysis. This involved the application of a z-score transformation prior to hierarchical clustering. All normalized chromatographic peak areas detected above background were included in the analysis. A Principal Component Analysis (PCA) was also conducted to understand the overall similarity/difference in the metabolome. To indicate the same samples across figures by the same markers, markers in PCA results are manually added to the result figures generated by Compound Discoverer. The original data figures are available in S5 Fig.

## 3 Results and discussion

### 3.1 Overall metabolome comparison

Overall, we identified 981 metabolites that could be detected by the procedure utilized in this study. Of those, 358 metabolites displayed statistically significant enrichment in two or more sample groups (i.e., large male, small male, large female, and small female). With these 358 metabolites, we further explored the differential presence of metabolites by specific pairs of comparison (Table 1). Among all compared pairs, the large and small male comparison indicated the largest number (140) of differentially enriched metabolites. Our results also suggest that higher level and possibly more diverse types of biochemical reactions may take place in large males (LM) when compared with the other three sample sets. Specifically, of those 140 differentially present metabolites, 75% (105 out of 140) were enriched in LM when compared with small males (SM), whereas 93% (28 out of 30) were enriched in LM when compared with large females (LF). Although both sex and body size are different, 80% (67 out of 84) were detected as significantly differentially enriched in LM when compared with small females (SF). Interestingly, comparison by sex indicated a much smaller number of enriched metabolites. Specifically, comparison between sex (LF vs. LM or SF vs. SM) indicated a total of 51 metabolites (30 metabolites for LF vs. LM and 21 for SF vs. SM), whereas 180 metabolites (140 from LM vs. SM and 40 from LF vs. SF) were identified from the comparison between body size. Unlike large males, large females did not indicate a significantly larger number of enriched metabolites when compared with the two other sample sets (SF or SM).

**Fig 1:**
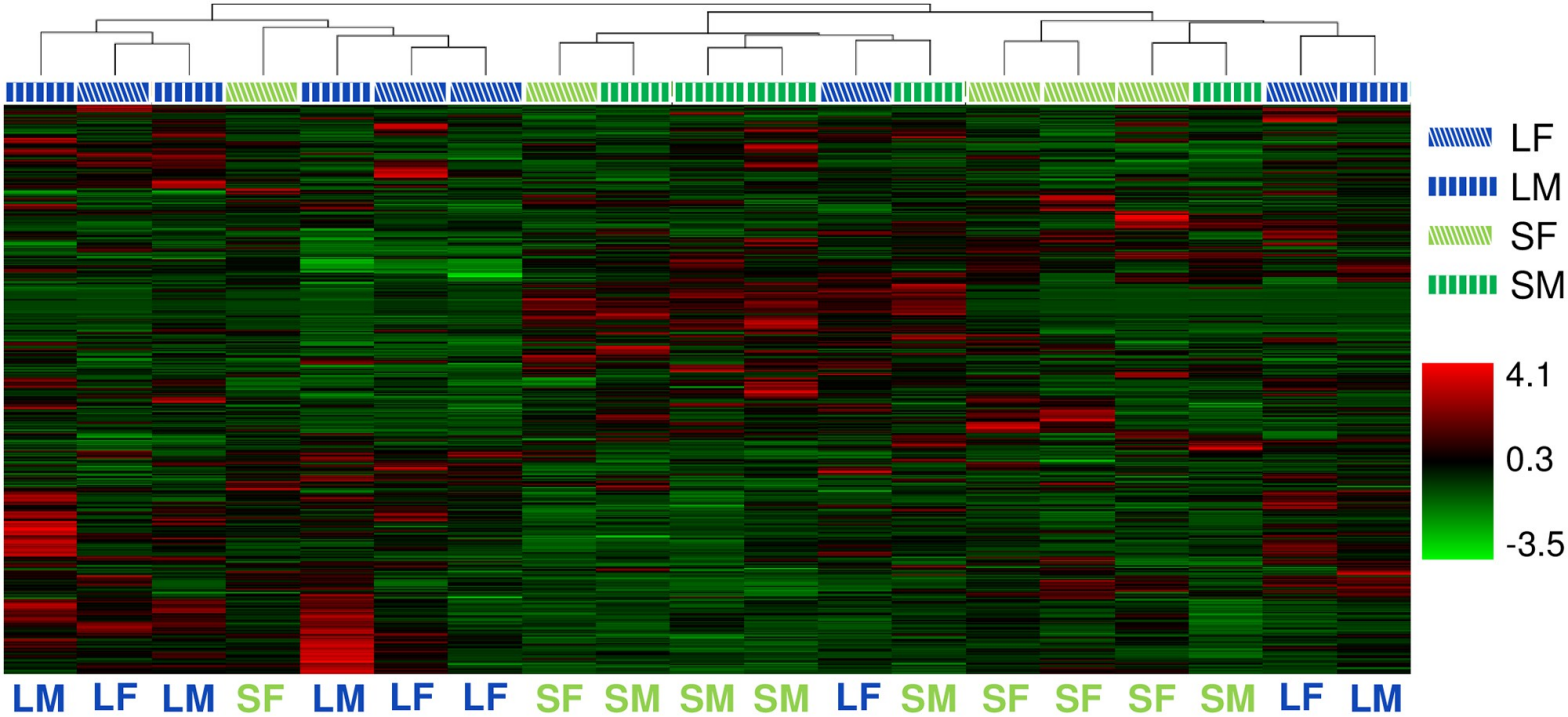
Hierarchical clustering for all samples used in this research. Blue and green represent large and small animals, respectively. Diagonal stripes and vertical stripes represent females and males, respectively. Note that one of large animal samples has been removed due to misplacement during sample preparation (see Methods section).

**Fig 2:**
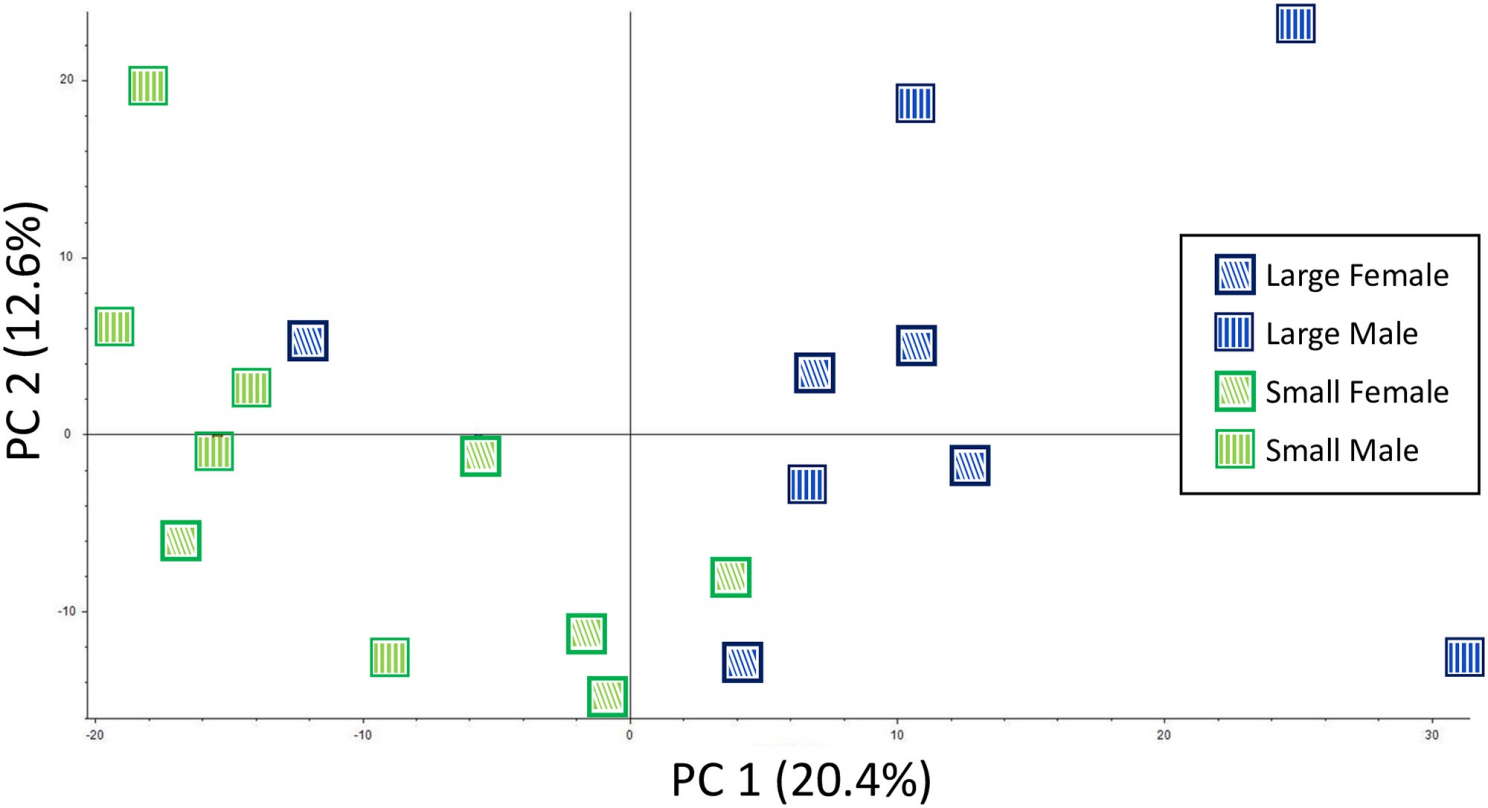
Principal component analysis of all samples used in this research. Blue and green represent large and small animals, respectively. Diagonal stripes and vertical stripes represent females and males, respectively. Note that one of large animal samples has been removed due to misplacement during sample preparation (see Methods section).

In order to examine the overall similarity or difference in the metabolome among samples, we conducted hierarchical clustering analysis (HCA) and principal component analysis (PCA) using all 19 individual beetle samples (see Materials and methods for details about sampling and metabolite detection). Both analyses implied that samples tend to cluster by size rather than sex (Figs 1 and 2). We then considered only one of these factors, namely sex or body size alone, to screen metabolites, and performed HCA and PCA with the same parameter settings. We detected a similar trend that supported the first set of results (S3 and S4 Figs). Specifically, PCA results clearly indicated the separation of small and large animals, whereas no grouping was seen when comparing the sexes. Moreover, HCA results indicated a dendrogram in which one clade is heavily enriched with large animals. However, samples from the same sex did not clearly cluster when only sex was considered to screen metabolites, again suggesting that the metabolome profile is relatively similar between sexes in a similar body size range. In contrast, previous transcriptomic studies using tissues of pupae (including horns) in different sexes and body sizes, suggested that sex is the most prominent factor that explains the variation of gene expression level [21]. Overall, our metabolic profiling using distinct traits (sex and body size) highlighted potential differences in the physiological processes among the samples we used. Particularly, higher and more diverse physiological activities may take place in large males over any of the other samples during the stage in which horn size is determined.

### 3.2 Candidate metabolites detected through pairwise comparisons

Since the overall comparison indicated a potential size effect in metabolomic differences, we further analyzed the data by only focusing on size (i.e., “large” samples consist of 4 LM and 5 LF samples, or “small” samples consist of 5 SM and 5 SF samples). In total, 107 metabolites showed significant presence in one category over the other. Of those, 24 chemical formulas were annotated in the ChemSpider database; however, these compounds could not be confirmed to a higher degree of confidence as none of the corresponding MS/MS spectra were matched in the mzCloud database. Then, we compared metabolite profiles by just focusing on sex (i.e., “male” samples consist of 4 LM and 5 SM samples, or “female” samples consist of 5 LF and 5 SF samples). In total, 30 metabolites indicated a significant presence in male or female. Of those 30, two had a match with mzCloud, D (or L)-Arginine and ornithine, which were more present in the male group than the female group by 1.26 times and 1.38 times, respectively. Although these two amino acids are critical components of the urea cycle in vertebrates, it is unclear if the same pathway is functional in insects, as they lack some enzymes in this cycle [22, 23].

To further identify candidate metabolites that may associate with polyphenic horn development, we attempted screening after the pairwise comparison. This analysis included both sex and size, and utilized the 358 metabolites that showed enrichment in two or more sample groups (Table 1). We obtained a list of moderate number (55), yet potentially critical, non-redundant metabolites that showed significant enrichment in certain samples and annotated by one or more databases (see Materials and methods). Of those, we focused on metabolites that showed significant differences between large males and other sample sets. Below, we list the metabolites we were able to annotate and discuss their potential significance in horn development. While most metabolites on this list were confirmed by mzCloud, we included one metabolite (D-xylonic acid) without a match in mzCloud due to its significant p-value and relatively large difference among the compounds we could identify (Table 2, S1 Table).

### 3.3 LM/SM or LF/SF pairwise comparison

We identified 10 metabolites that were shared between the LM/SM and LF/SF comparisons. None of these 10 had a match in mzCloud, however, D-xylonic acid was found to be significantly higher in small males than large males by 5.78 times and 3.73 times greater in small females than large females. This compound is synthesized by oxidizing xylose that is derived from hemicellulose, a major component of plant cell walls [24]. Animals usually require gut microbes to metabolize plant material. Since all beetle larvae were reared under the same conditions with the same food, it is likely that all animals used in this study harbored similar gut microbiomes. Thus, accumulation of xylonic acid in small animals may not be attributed to the differential activity of gut microbes, rather it may indicate that the resource is not fully metabolized due to a relatively slower overall metabolism (cell activity) in small animals.

### 3.4 Large/small male comparison

We sought compounds that were found to have significantly different enrichment within the large and small male comparison as they could potentially reflect direct differences in horn development. Of 140 compounds we detected as significantly different (Table 1), 30 indicated a match in ChemSpider. Of these 30, five metabolites were annotated by mzCloud. With these five, we compared all other possible pairwise combinations that involve large males (i.e., LM/LS, LM/LF, LM/SF) and identified three compounds that are only present in the LM/SM comparison, while the other two showed enrichment in large males versus the other sample groups (all of SM, LF, and SF). The former three include N-acetyl-DL-glutamic acid, glycerol 3-phosphate, and citric acid (shaded metabolites in Table 2).

Citric acid was detected in both male types and was enriched 1.5 times more in large males than in small males. It is a major intermediate in the citric acid cycle (also known as TCA or Krebs cycle) that generates NADH which further links to ATP generation. Therefore, the increase in citric acid in large males may represent high cellular activity or rapid cell division.

Glycerol-3-phosphate (G3P) was indicated as significantly greater in large males than small males by 2.1 times. In *Drosophila* and mammals, G3P can be utilized to maintain redox balance (NAD^+^ and NADH ratio) in cells that affects ATP production. *Drosophila* mutant larvae that lack the key enzyme in this cycle, lactate dehydrogenase (LDH), can develop normally because the increased level of G3P compensates for the expected low-energy status caused by the lack of the *Ldh* gene [25]. Thus, by analogy, the higher level of G3P in large male *O*. *taurus* may indicate a lower activity of *Ldh*. Intriguingly, our previous transcriptome study also showed that the *Ldh* level was lower in the head horn tissue of large male pupa [21] (note that the animals’ stage used is different from our current study). Alternatively, G3P can be involved in fatty acid synthesis rather than mobilization. When fatty acids are synthesized in conditions of excess energy input, they are esterified to G3P and stored as triglycerides [26]. In this case, the enrichment of G3P in large males may reflect a higher nutritional condition which would enable them to store more energy than their small male counterparts. Since the animals we used in this research were in their prepupal stage, a stage where extensive tissue degradation and reconstruction associated cell division/growth/death take place, this scenario may be less likely. While we reserve our conclusion on the cause of enriched G3P only in large males, high citric acid presence likely indicates heightened cellular activity such as cell growth in general and possibly large horn development in particular.

Unlike the two metabolites above, N-acetyl-DL-glutamic acid (N-acetylglutamate, NAG) was identified as a compound that was significantly enriched in small males versus large males by 1.82 times. Although the biochemical function of NAG in dung beetles is not known, it is known that a high-fat diet in *Drosophila melanogaster* lowers the level of amino acids including glutamate, which can be converted to NAG [27]. This may imply relatively higher levels of fat accumulation in large males.

Previous metabolome studies used other insects to highlight metabolite differences in gonads between males and females or ejaculate of males in different body sizes. These studies identified potential male (sperm)-specific metabolites or metabolites in male ejaculates [28–30]. Studies of potential sperm competition in genus *Onthophagus* provided insights into evolutionary and ecological significance of testis-horn size relationships [31, 32]. Moreover, a trade-off between male genitalia development and horn size determination has been reported using *O*. *taurus* [33]. Thus, we sought candidate metabolites that are specifically detected by the male-female comparison or the large-small male comparison (see above). Our criteria did not identify metabolites other than D-xylonic acid. This may suggest the metabolic condition in prepupal hemolymph is relatively well-regulated and stable due to homeostasis. By analogy, metabolites identified in the current study might be strong candidates representing sex- or morph-specific biological processes.

Overall, our comparative analyses identified some key metabolites in critical biochemical pathways for cellular activities and highlighted significant differences between large and small males.

### 3.5 Large male/other samples

As an alternative way to discover metabolites that may represent biochemical processes critical for the horn development, we compared metabolomic profiles and screened ones that were enriched in the large male group over any other group (i.e. large female, small female, small male). Of the 264 metabolites combined (LM/SM, LM/LF, and LM/SF, Table 1), seven met the above criteria and had a match in ChemSpider. Of those seven, we further confirmed two metabolites (glutamine and N-acetyl-L-glutamine) using mzCloud (Table 2).

D-glutamine was identified as a compound that is enriched in large males by 1.57 times, 1.48 times, and 1.40 times more than small males, small females, and large females, respectively. Glutamine can be converted to glutamate which can then be further converted to α-ketoglutarate through a process known as glutaminolysis, which can fuel citric acid cycle [34] that is likely to be active in large males.

N-acetyl-L-glutamine (acetylglutamine, aceglutamide), a derivative of glutamine, showed 2.36 times, 2.87 times, and 2.04 times enrichment in large males over small males, small females, and large females, respectively. Although the physiological function of this metabolite is not clear in insects, in humans, it is known to provide protection to organs in patients under protein energy malnutrition, which can occur when individuals experience highly stressful nutritional conditions such as a severe malnutrition or secondarily caused by cancer [35, 36].

On one hand, the large/small male comparison identified biologically very significant and relevant, thus intuitive, compounds such as citric acid. On the other hand, the comparison between large males and multiple samples shed light on rather unfamiliar compounds in the context of beetle horn development. Thus, our results can provide a new scope on the biological processes that transcriptomic studies did not highlight. Here, we propose that amino acids, particularly glutamine and its derivatives may be key molecules in beetle development, or more specifically in males that develop large horns. The mechanisms by which these compounds affect the development of large males are still unclear. However, we speculate unusually rapid cell proliferation and growth in large male’s head (horn) may be associated with enrichment of these compounds.

## 4 Concluding remarks

From genetic content (genome) or expressed genes (transcriptome), to actual functioning molecules (proteome), advances in molecular biology have provided researchers with information on non-model organisms through so called -omics approaches. Here, we report, to our knowledge, the first metabolomic dataset highlighting the physiological profiles of dung beetles. In this work, we aim to identify key metabolites that may reflect the physiological differences between males that develop horns in distinct sizes.

Through transcriptome studies, we identified genes that are not sensitive to nutritional input in their original context. For instance, the sex determination gene *doublesex* (*dsx*) is expressed more in the horns of large males, suggesting *dsx* might have been sensitized to changes in certain metabolites during the horn development. We were able to detect differentially enriched metabolites between large and small males, which may offer a new opportunity towards understanding the interaction of genetic processes, such as sex determination and physiological processes such as energy production. For instance, the overall pattern of metabolite enrichment suggests that body size rather than sex reflects the differences in metabolomic profiles. In contrast, the transcriptome study indicated that sex was a stronger factor than body size in explaining gene expression variation [21]. Further integrated analyses of the transcriptome and metabolome across developmental stages (e.g., third instar larva and/or pupa, which was used in almost all *O*. *taurus* transcriptome studies) and different tissues in different body sizes would provide insight into the complex and systemic aspects of polyphenic trait development.

Specific metabolites discovered in this study suggest vigorous cellular processes such as cell division or growth take place in large males. Interestingly, some compounds identified in large males (e.g., citric acid and glycerol-3-phospate) were not identified in large/small female comparison. Since the horn primordia in males rapidly develop and respond to nutritional input in the stage we studied, we suspect these compounds are highly utilized/mobilized in large males during the horn development. Furthermore, we identified metabolites associating with glutamine/glutamate metabolism, which was rather unexpected. In the past, transcriptome studies unexpectedly identified some “horn polyphenism-related genes” such as genes in the Hedgehog pathway after their function was confirmed by RNAi [12]. We consider the glutamine/glutamate metabolism may be a process we could have missed unless we conducted the metabolomic study.

Studying the metabolome in non-model organisms still requires efforts in establishing methods in metabolite extraction as well as metabolite identification through database searches. Our current results were obtained by using a method that may not be optimized for certain compounds. Establishing methods for more focused compound extraction (e.g., hormones, sugars, etc.) would enrich our understandings in the physiological basis of horn polyphenism. Pathway enrichment analyses that require existing evidence in databases would further identify key physiological processes in polyphenic horn development. More generally, when the trait development associates with nutritional (physiological) changes, integrating the knowledge obtained from both the transcriptome and metabolome studies can lead to the discovery of new and critical biological processes that each study alone may not highlight.

## Supporting information

S1 Fig Frontal view (top row) and lateral view (bottom row) of *O*. *taurus*. Male in different body size with distinct difference in the horn size and shape along with female are shown.
(PPTX)

S2 Fig Distribution of sample body masses used in this study. (PPTX)

S3 Fig Results of HCA by size (A) or sex (B) alone. (PPTX)

S4 Fig Results of PCA by size (A) or sex (B) alone. (PPTX)

S5 Fig Original figures of PCA results generated by Compound Discoverer. (A) all 19 samples, (B) large (LM+LF) and small (SM+SF) comparison, and (C) male (LM+SM) and female (LF+SF) comparison.
(PPTX)

S1 Table Compounds that showed significant difference between any given pairwise comparison. All compounds including unannotated (no MS/MS match, hence only the chemical formula) ones are also listed.
(XLSX)
