# Supplementary figures and images for: Comparative metabolomic analysis of polyphenic horn development in the dung beetle Onthophagus taurus
Source: PLoS One. 2022 Mar 17;17(3):e0265222. doi: 10.1371/journal.pone.0265222 (PMC8929603; doi:10.1371/journal.pone.0265222)

## Slide 1
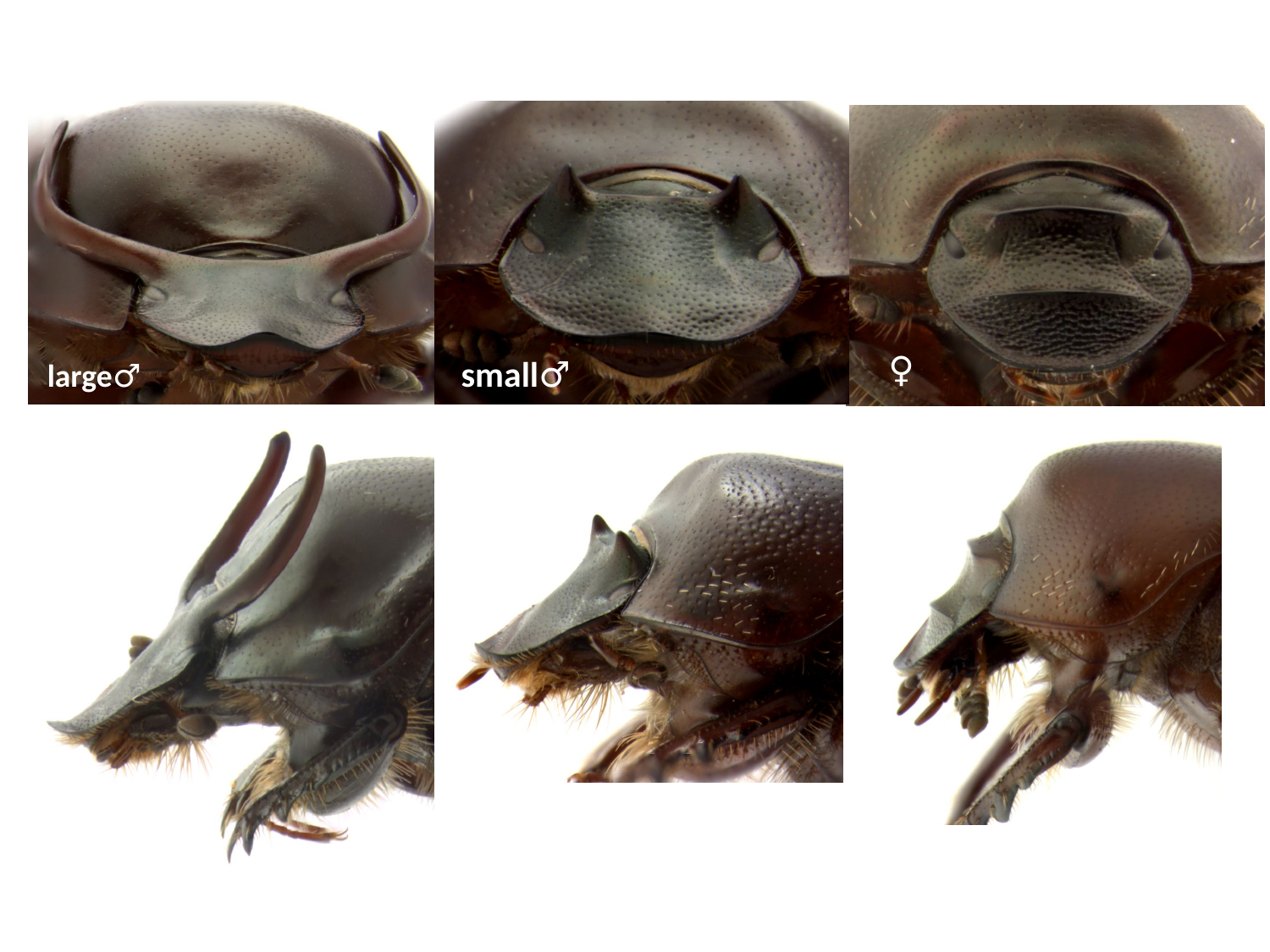

♀
 small♂
large♂

Supplement: S1 Fig — Male in different body size with distinct difference in the horn size and shape along with female are shown. (PPTX) [file pone.0265222.s001.pptx]

## Slide 1
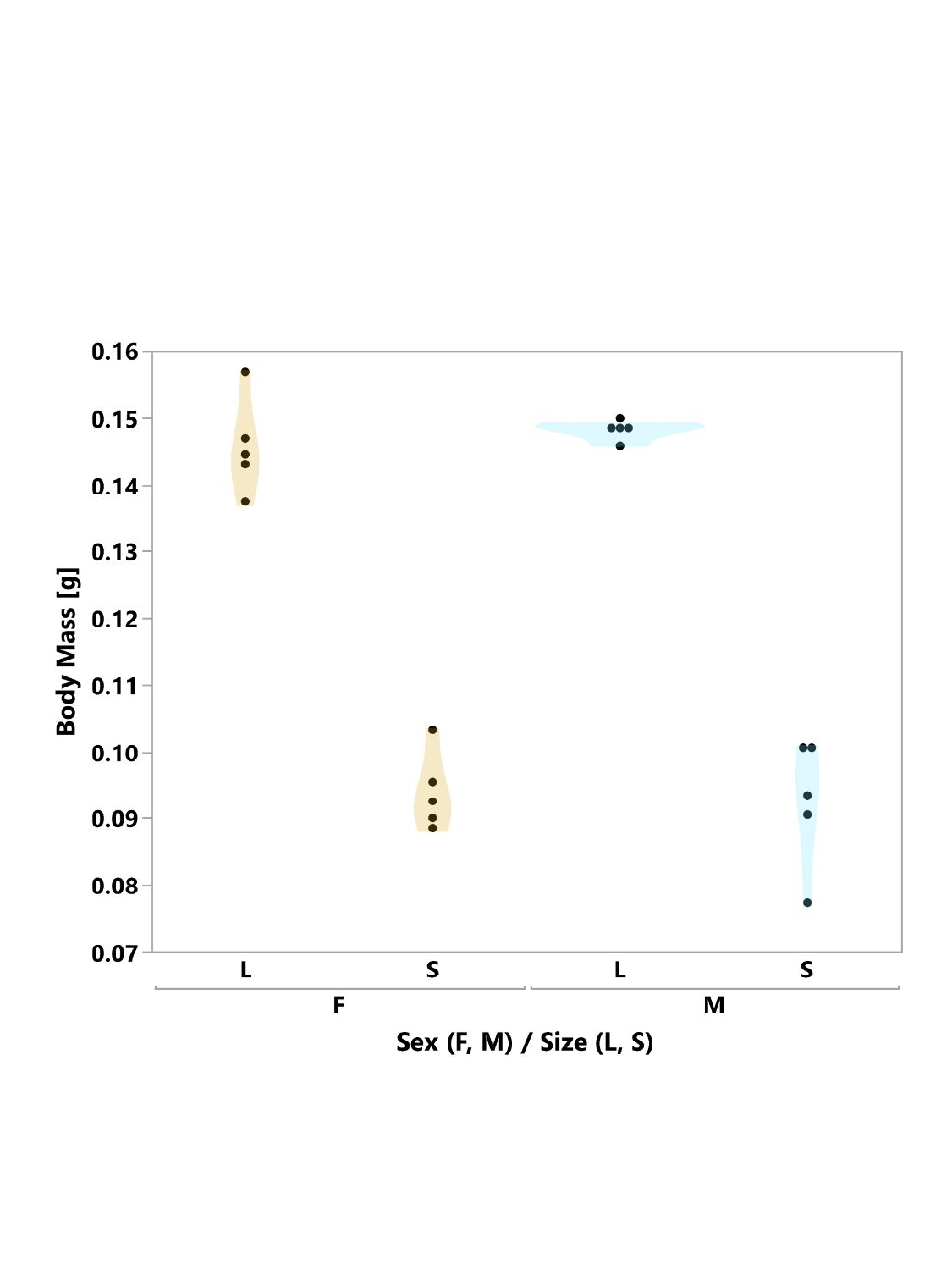

Supplement: S2 Fig — (PPTX) [file pone.0265222.s002.pptx]

## Slide 1
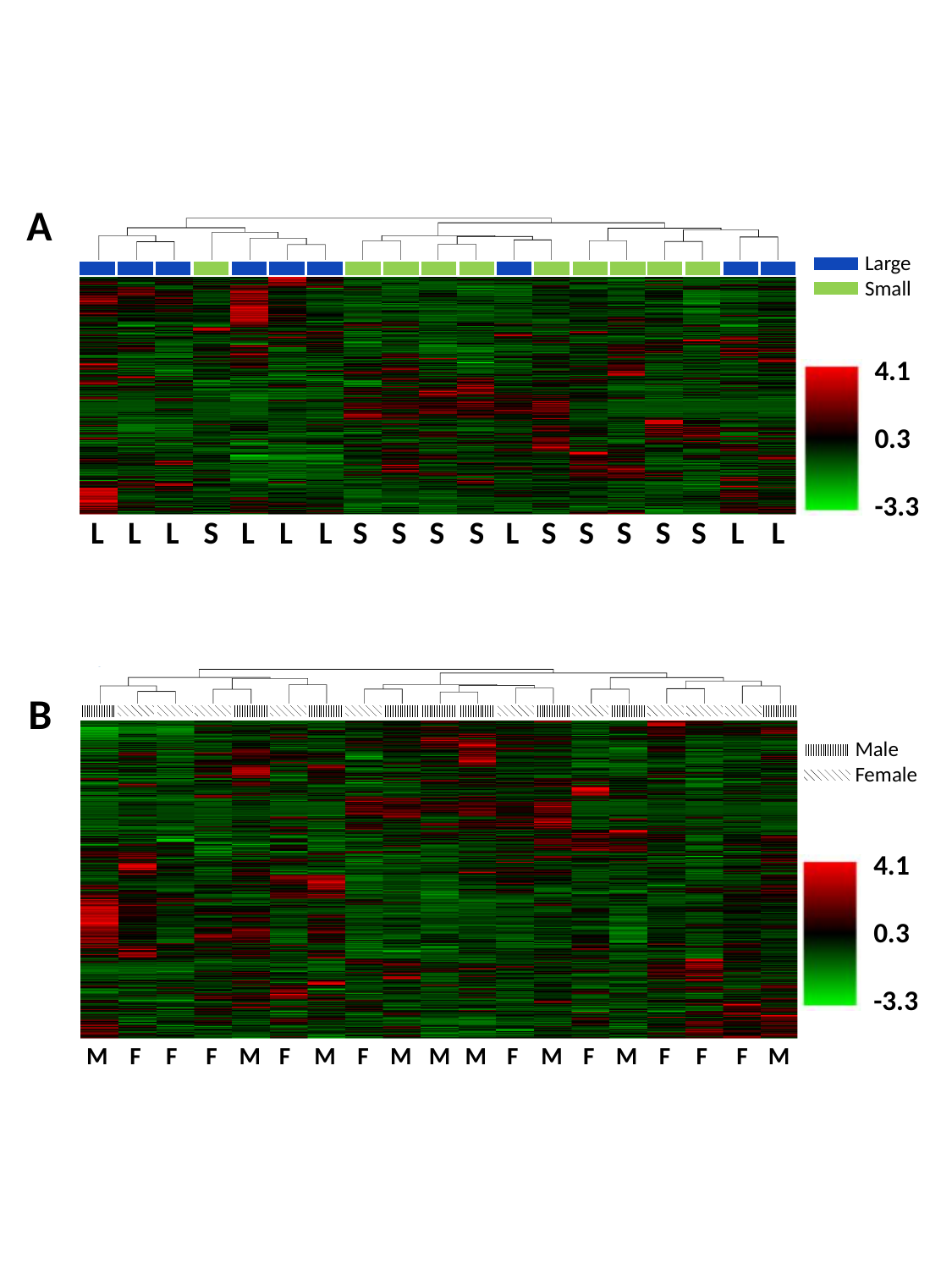

A
Large
Small
4.1
0.3
-3.3
L
L
L
S
L
L
L
S
S
S
S
L
S
S
S
S
S
L
L
B
Male
Female
4.1
0.3
-3.3
M
F
F
F
M
F
M
F
M
M
M
F
M
F
M
F
F
F
M

Supplement: S3 Fig — (PPTX) [file pone.0265222.s003.pptx]

## Slide 1
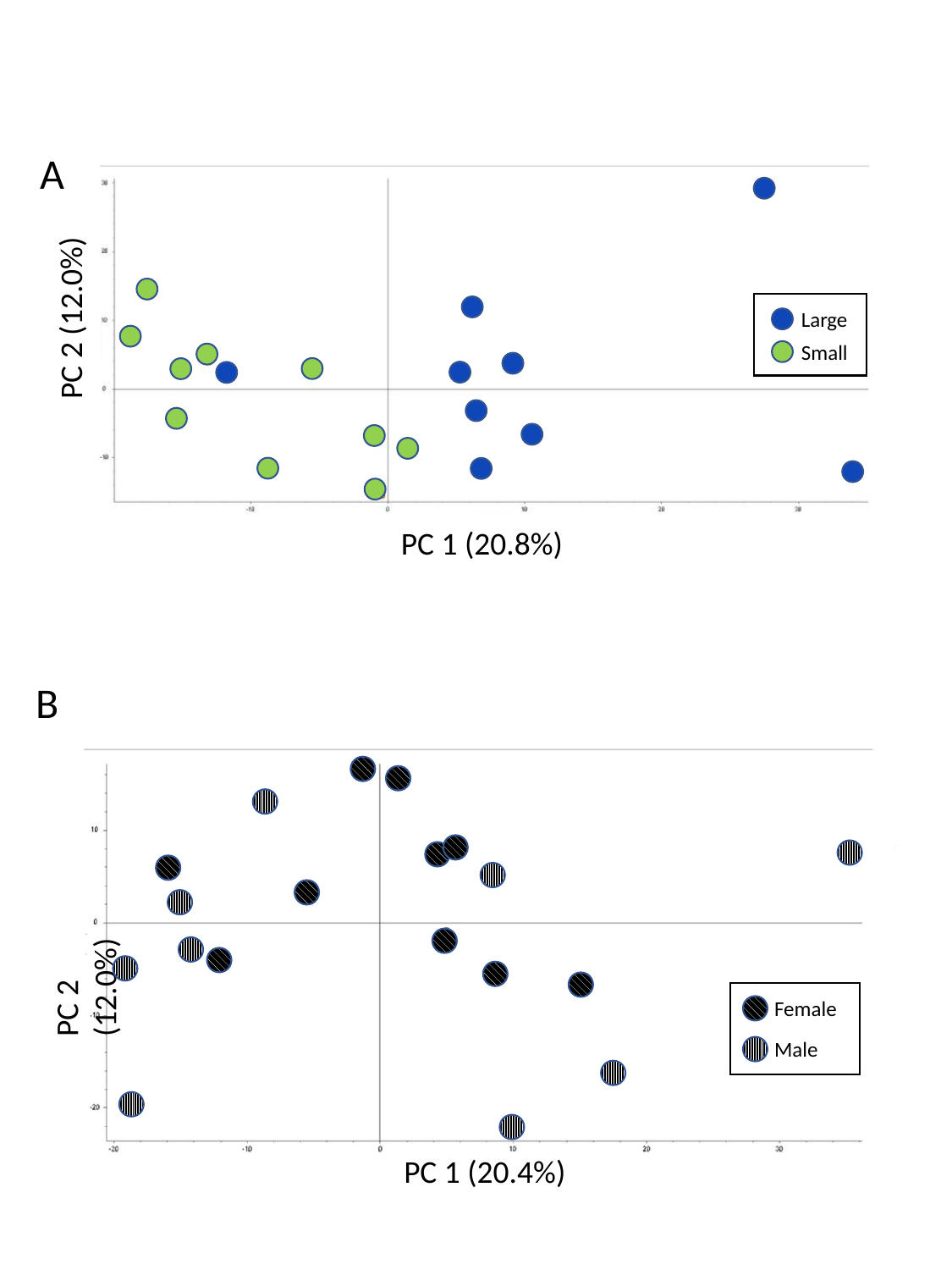

A
PC 2 (12.0%)
Large
Small
PC 1 (20.8%)
B
PC 2 (12.0%)
Female
Male
PC 1 (20.4%)

Supplement: S4 Fig — (PPTX) [file pone.0265222.s004.pptx]

## Slide 1
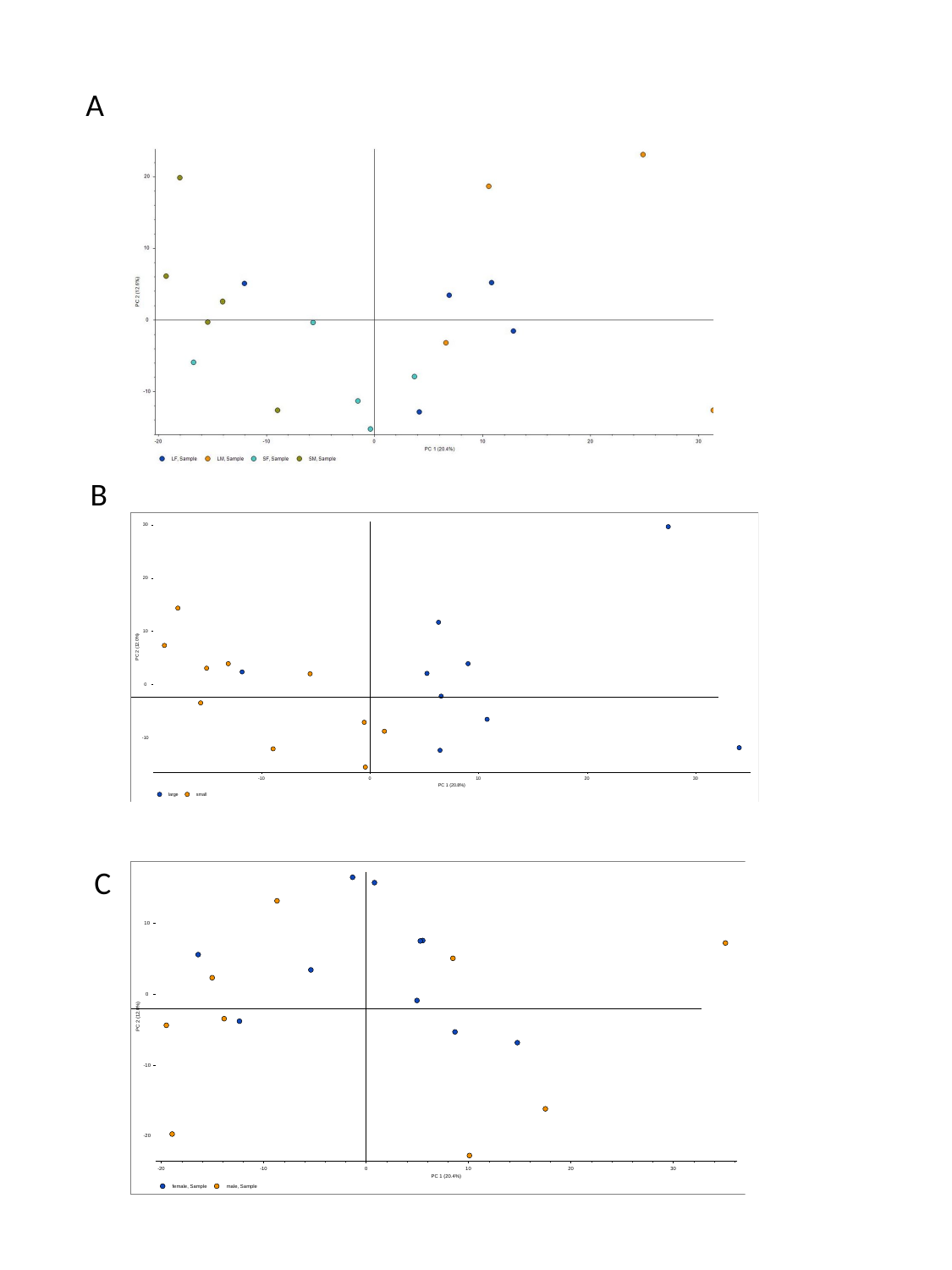

A
B
C

Supplement: S5 Fig — (A) all 19 samples, (B) large (LM+LF) and small (SM+SF) comparison, and (C) male (LM+SM) and female (LF+SF) comparison. (PPTX) [file pone.0265222.s005.pptx]
